# Development and validation of a prediction model for mortality in critically ill COVID-19 patients

**DOI:** 10.3389/fcimb.2024.1309529

**Published:** 2024-06-24

**Authors:** Xiaoxiao Sun, Jinxuan Tang, Jun Lu, Hui Zhang, Cheng Li

**Affiliations:** ^1^ Department of Critical Care Medicine, Shanghai Key Laboratory of Anesthesiology and Brain Functional Modulation, Clinical Research Center for Anesthesiology and Perioperative Medicine, Translational Research Institute of Brain and Brain-Like Intelligence, Shanghai Fourth People's Hospital, School of Medicine, Tongji University, Shanghai, China; ^2^ Department of Anesthesiology and Perioprative Medicine, Shanghai Key Laboratory of Anesthesiology and Brain Functional Modulation, Clinical Research Center for Anesthesiology and Perioperative Medicine, Translational Research Institute of Brain and Brain-Like Intelligence, Shanghai Fourth People's Hospital, School of Medicine, Tongji University, Shanghai, China

**Keywords:** COVID-19 infection, critically ill, prediction model, propensity matching scores, logistic regression

## Abstract

**Background:**

Early prediction of prognosis may help early treatment measures to reduce mortality in critically ill coronavirus disease (COVID-19) patients. The study aimed to develop a mortality prediction model for critically ill COVID-19 patients.

**Methods:**

This retrospective study analyzed the clinical data of critically ill COVID-19 patients in an intensive care unit between April and June 2022. Propensity matching scores were used to reduce the effect of confounding factors. A predictive model was built using logistic regression analysis and visualized using a nomogram. Calibration and receiver operating characteristic (ROC) curves were used to estimate the accuracy and predictive value of the model. Decision curve analysis (DCA) was used to examine the value of the model for clinical interventions.

**Results:**

In total, 137 critically ill COVID-19 patients were enrolled; 84 survived, and 53 died. Univariate and multivariate logistic regression analyses revealed that aspartate aminotransferase (AST), creatinine, and myoglobin levels were independent prognostic factors. We constructed logistic regression prediction models using the seven least absolute shrinkage and selection operator regression-selected variables (hematocrit, red blood cell distribution width-standard deviation, procalcitonin, AST, creatinine, potassium, and myoglobin; Model 1) and three independent factor variables (Model 2). The calibration curves suggested that the actual predictions of the two models were similar to the ideal predictions. The ROC curve indicated that both models had good predictive power, and Model 1 had better predictive power than Model 2. The DCA results suggested that the model intervention was beneficial to patients and patients benefited more from Model 1 than from Model 2.

**Conclusion:**

The predictive model constructed using characteristic variables screened using LASSO regression can accurately predict the prognosis of critically ill COVID-19 patients. This model can assist clinicians in implementing early interventions. External validation by prospective large-sample studies is required.

## 1 Introduction

Since the 2020 coronavirus disease (COVID-19) pandemic, another circulation of the virus had occurred in Shanghai, China. The COVID-19 pandemic will inevitably have a major effect on the global economy and healthcare (Lim et al., 2021). Patients with severe COVID-19 consume most healthcare resources and have a high mortality rate. As shown in a previous study, the mortality rate of critically ill COVID-19 patients is approximately 38.7%, which is similar to the results of a meta-analysis on the mortality of critically ill COVID-19 patients (40–50%) (Armstrong et al., 2020). Another previous study (Huang et al., 2020) showed that critically ill COVID-19 patients had a higher risk of clinical characteristics (e.g., age, dyspnea, and lymphocyte count <1.5×10^9^/L) and multiple system organ complications (acute cardiac injury, acute respiratory distress syndrome, acute kidney injury, and shock). Mortality rate in critically ill COVID-19 patients was significantly higher than in non-critically ill COVID-19 patients, even when they received more major interventions. Therefore, early detection and efforts to explore effective therapies for critically ill COVID-19 patients are critical. Early prediction of mortality risk may help optimize treatment, reallocate healthcare resources, and reduce mortality in critically ill COVID-19 patients (Lerner et al., 2008; Song et al., 2020).

Recently, several risk models for predicting death in COVID-19 patients have been published. These studies incorporated variables from different perspectives to build predictive models, such as the creation of a new scoring system or the development of models through regression analysis; all models had good predictive performance (Bello-Chavolla et al., 2020; Hu et al., 2020; Ji et al., 2020; Knight et al., 2020; Berenguer et al., 2021). However, most studies have examined the entire COVID-19-infected population, regardless of disease progression. These prediction models failed to consider the clinical use of specific models. A previous study found that the myoglobin level at intensive care unit (ICU) admission was an independent risk factor for death in critically ill COVID-19 patients and was linearly associated with mortality risk. Therefore, this study aimed develop a mortality prediction model for critically ill COVID-19 patients. We used least absolute shrinkage and selection operator (LASSO) regression and logistic regression analyses to build the mortality prediction models to assist clinicians in identifying and implement early interventions for critically ill COVID-19 patients at risk of death due to critical infections.

## 2 Study design and methods

### 2.1 Patients

This study retrospectively analyzed the clinical data of critically ill COVID-19 patients who were hospitalized in the ICU of a university hospital between April and June 2022. Patients were recruited based on the following inclusion criteria: (1) patients meeting the diagnostic criteria of the Diagnostic Protocol for Novel Coronavirus Pneumonia (Trial 10th Edition) issued by the National Health and Wellness Commission and admission to the ICU; (2) those who met any of the following diagnostic criteria for critically ill COVID-19 patients: (a) respiratory failure requiring mechanical ventilation, (b) shock, and (c) organ failure requiring intensive care monitoring; and (3) age ≥18 years. The exclusion criteria were as follows: (1) patients whose family members voluntarily signed a consent form to withhold active resuscitation or other serious underlying diseases that led to death, (2) pregnant women, and (3) lack of laboratory markers within 48 h of ICU admission.

### 2.2 Data collection

Clinical and laboratory information of the participants was collected during clinical training from the electronic medical record system. It included the following details: (1) general data (sex and age); (2) clinical admission data (underlying disease, referral, vaccination, days of hospitalization, and prognosis); and (3) laboratory markers within 48 h of ICU admission (routine blood count, liver and kidney function parameters, myocardial markers, coagulation function parameters, inflammatory indices, and inflammatory factors). These laboratory markers were divided into two categories based on whether they were within the normal range.

### 2.3 Statistical analyses

Propensity matching scores were used to reduce the effect of observed confounding factors. Normally distributed continuous variables were expressed as mean ± standard deviation, whereas non-normally distributed continuous variables were presented as median ± interquartile range. Categorical variables are expressed as percentage (%). Normally distributed continuous variables were analyzed using the Student t-test, whereas non-normally distributed variables were analyzed using the Mann–Whitney U test. Categorical variables were assessed using the chi-square test. Statistical analyses were performed using R, version 4.2.2 (the R Project for Statistical Computing). A P-value <0.05 was considered statistically significant.

Characteristic variables were screened using the LASSO logistic regression analysis, and univariate and multivariate logistic regression analyses were used to assess the association between screened variables and patient prognosis. A predictive model was built using logistic regression analysis and visualized using a nomogram. Calibration and receiver operating characteristic (ROC) curves were used to estimate the accuracy and predictive value of the model. Decision curve analysis (DCA) was used to examine the value of the model for clinical intervention.

## 3 Results

### 3.1 Patient characteristics

Clinical data were collected from 137 critically ill COVID-19patients, of whom 84 survived (survival group) and 53 died (death group). Baseline information and biochemical test results for all patients are shown in **Supplementary Table 1** (**Supplementary Table 1**). The baseline data of the patients showed differences in hypertension, diabetes mellitus, and stroke between the two groups (P<0.05). To eliminate the effect of underlying diseases, we matched deceased and surviving patients at a 1:1 ratio using propensity score matching, resulting in 43 surviving and 43 deceased patients in this study. Statistically significant differences were observed in the red blood cell distribution width-standard deviation (RDW SD), procalcitonin, aspartate aminotransferase (AST), creatinine, potassium, troponin I, and myoglobin values. The results for the matched patients are shown in **Supplementary Table 2** (**Supplementary Table 2**).

### 3.2 Selection of characteristic variables

**Figure 1:**
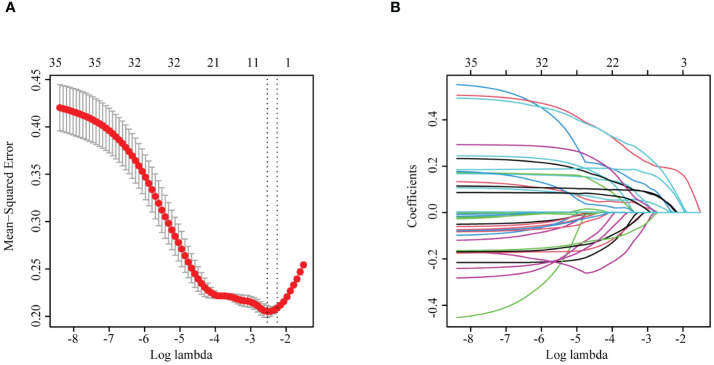
**(A)** LASSO cross-validation curve. **(B)** Plot of the coefficients of the LASSO regression model variables. LASSO, least absolute shrinkage and selection operator.

**Figure 2:**
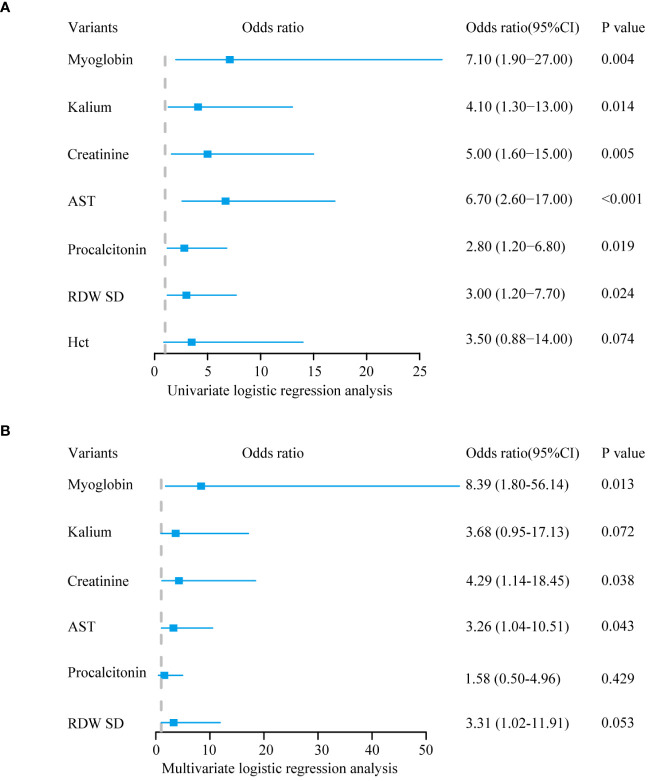
Logistic regression analysis. **(A)** The outcome of univariate logistic regression analysis. **(B)** The outcome of multivariate logistic regression analysis.

To identify the characteristic variables in the death group, we analyzed the variables using LASSO logistic regression analysis, and seven variables (hematocrit (Hct), RDW SD, procalcitonin, AST, creatinine, potassium, and myoglobin) were finally screened (**Figures 1A, B**). Univariate and multivariate logistic regression analyses were used to evaluate the effect of the seven variables on mortality to further identify the clinical outcomes associated with mortality. The analyses showed that three variables (creatinine, AST, and myoglobin) in the abnormal range were independent risk factors for mortality (**Table 1**, **Figure 2**).

**Table 1:** Results of univariate and multivariate logistic regression analyses with variants selected by LASSO regression analysis for patients.

Variants	Univariate logistic regression		Multivariate logistic regression	
	OR (95% CI)	P-value	OR (95% CI)	Adjusted P-value
Hct	3.50 (0.88–14.00)	0.074	–	–
RDW_SD	3.00 (1.20–7.70)	0.024*	3.31 (1.02–11.91)	0.053
Procalcitonin	2.80 (1.20–6.80)	0.019*	1.58 (0.50–4.96)	0.429
AST	6.70 (2.60–17.00)	<0.001*	3.26 (1.04–10.51)	0.043*
Creatinine	5.00 (1.60–15.00)	0.005*	4.29 (1.14–18.45)	0.038*
Potassium	4.10 (1.30–13.00)	0.014*	3.68 (0.95–17.13)	0.072
Myoglobin	7.10 (1.90–27.00)	0.004*	8.39 (1.80–56.14)	0.013*

^*^p<0.05.

LASSO, least absolute shrinkage and selection operator; OR, odds ratio; CI, confidence interval; Hct, hematocrit; RDW_SD, red blood cell distribution width-standard deviation; AST, aspartate aminotransferase.

### 3.3 Establishment of the prediction models

**Figure 3:**
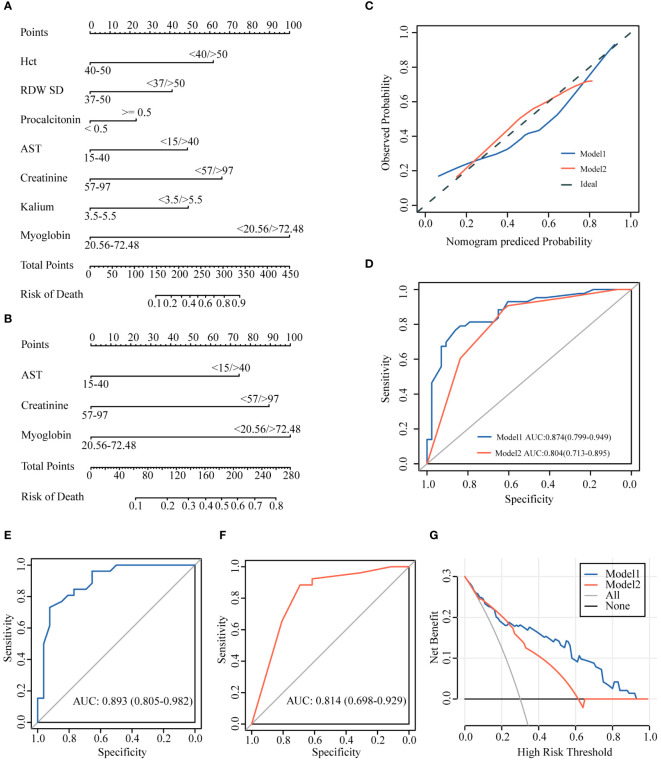
Establishment of the prediction models. **(A)** Model 1 based on 7 variants. **(B)** Model 2 based on 3 variants. **(C)** Calibration curves. **(D)** AUCs of the two models (AUC of Model1: 0.874; AUC of Model2: 0.804). **(E, F)** AUCs of internal validation using 60% data (AUC of Model1: 0.893; AUC of Model2: 0.814). **(G)** DCA curve showing the benefit from the models in the clinical setting. AUC, area under the curve; DCA, decision curve analysis.

We constructed logistic regression models using the seven variables after LASSO logistic regression analysis (Model 1) and three independent risk factors for death after LASSO logistic regression and univariate and multivariate logistic regression analyses (Model 2). The two models were visualized using a nomogram (**Figures 3A, B**). The model was validated using calibration curves, and the actual predictions of the two models were similar to the ideal predictions, demonstrating a good agreement between the predictions and observations (**Figure 3C**).

### 3.4 Model performance and internal validation

The predictive ability of the models was evaluated using ROC curve analysis, and the area under the curve (AUC) indicated that both models had good predictions (Model 1: AUC=0.874 and Model 2: AUC=0.804). Moreover, the predictive accuracy of Model 1 was higher than that of Model 2 (**Figure 3D**). We selected 60% of the data to validate the model predictions. The results showed that both models had a good predictive ability [Model 1: AUC=0.893 (0.805–0.982) and Model 2: AUC=0.814 (0.698–0.929)], although Model 1 was better than Model 2 in the accuracy of predicting outcomes in critically illCOVID-19 patients (**Figures 3E, F**).

### 3.5 Clinical benefits

We used DCA to estimate whether patients could benefit from the models in clinical practice. The intervention was beneficial for patients according to Model 1 when the threshold probability was 0.1–1, and the intervention according to Model 2 was beneficial to patients if the threshold probability was 0.1–0.6. When the threshold probability was >0.22, the clinical intervention effect based on Model 1 was better than that based on Model 2 (**Figure 3G**).

## 4 Discussion

This study aimed to develop a mortality prediction model for critically ill COVID-19 patients, and we found that the predictive model constructed using characteristic variables screened using LASSO regression can accurately predict the prognosis of critically ill COVID-19 patients. In recent years, various models have been developed to predict the mortality risk in COVID-19 patients. The models developed by Bo et al (Chen B. et al., 2021). and Cheng et al. (2022) were prediction models for mortality risk in severely/critically ill patients and were validated to show that the models had good predictive ability in all cases. However, owing to missing data, no other prognostic laboratory indicators such as AST and lactate dehydrogenase (LDH) were collected in these two studies. In contrast, the mortality prediction models developed by Zeng et al. (2021), Chen et al (Chen H. et al., 2021), and Li et al. (2021) did not differentiate the disease severity. Our study was conducted in critically ill COVID-19 patients to establish a model that can predict mortality risk in such patients, who consume the most healthcare resources and require optimization of treatment decisions and allocation of limited healthcare resources. Owing to the small sample size included in this study and many independent variables and covariates between different variables, LASSO regression analysis was used to screen potential independent variables. Predictive models were developed directly based on the selected variables (Model 1) and through univariate and multivariate logistic regression analyses (Model 2). Model 1 consisted of seven variables (Hct, RDW SD, procalcitonin, AST, creatinine, potassium, and myoglobin), whereas Model 2 consisted of three variables (AST, creatinine, and myoglobin). Through internal validation, we further validated the two models, showing that both had good model effects and good predictive performance. According to the AUC, the prediction of Model 1 was better than that of Model 2.

Several studies have reported independent risk factors for the prognosis of COVID-19 patients. Fathalla et al. (2022) reported that COVID-19 patients with comorbid hypertension and/or diabetes mellitus had more severe disease and poorer prognosis and the levels of ultrasensitive C-reactive protein (CRP), D-dimer, neutrophil to lymphocyte ratio, and LDH at admission were independent risk factors for patient prognosis. A retrospective analysis by Donoso-Navarro et al. (2021) found that COVID-19 patients had more severe disease and prognosis. Studies have also shown that age, LDH, interleukin-6, and lymphocyte count are independent predictors of patient mortality risk. A case-control study by Pan et al. (2020) in China included 124 critically ill COVID-19 patients. The findings showed that peripheral oxygen saturation, lymphocyte count, CRP, procalcitonin, and LDH values at admission were independent mortality risk factors critically ill COVID-19 and could be used as independent predictors of clinical prognosis. In contrast to previous studies, the present study showed that abnormal levels of myoglobin, creatinine, and AST within 48h of ICU admission were independent risk factors for mortality in critically ill COVID-19 patients. Angiotensin-converting enzyme 2 (ACE2) is expressed in endothelial and smooth muscle cells of almost all organs, and ACE2-induced invasion in COVID-19 may lead to infection and impairment of multiple organ functions with subsequent activation of immune responses and cytokine inflammatory storms, further exacerbating organ dysfunction (Xu et al., 2020). Meanwhile, non-steroidal anti-inflammatory drugs, antivirals, antibiotics, and herbal medicines used to relieve clinical symptoms such as fever can also cause liver and kidney damage. Therefore, the AST and creatinine levels in these patients were abnormal. Furthermore, various conditions such as severe infection, systemic inflammation, and organ failure, can cause increase myoglobin levels, which correlate with disease severity and prognosis (Kurt-Mangold et al., 2012). COVID-19 leads to hypoxia and systemic inflammatory storms (Hendgen-Cotta et al., 2014; Han et al., 2020; He et al., 2020; South et al., 2020), causing nonspecific damage to multiple organs and further increasing myoglobin levels. Critically ill patients have a lower oxygenation index, more severe hypoxia, and higher myoglobin levels than their counterparts.

In this study, a predictive model for assessing ICU survival in critically ill COVID-19 patients was constructed using clinical information and test results at ICU admission and was internally validated as a good predictor of outcomes after ICU admission in this patient population. In addition, screening variables using LASSO regression analysis has the advantage of simplifying statistical models, reducing multivariate covariance, and improving model accuracy. These models can help physicians in the ICU identify patients at high risk of premature death, and timely intervention will lead to appropriate treatment strategies, thereby potentially improving patient prognosis. An accurate prognostic assessment is essential for the rational allocation of limited medical resources. Nevertheless, this study has some limitations. First, it was a single-center, retrospective study with a small sample size. Thus, external validation by prospective, large-sample studies is required. Second, laboratory-accessible indicators change with disease progression, and retrospective studies could not incorporate dynamic changes in indicators into the model for analysis. Lastly, the virus type included in this study was the Omicron BA.2 variant; thus, whether our results are applicable to infection with other variant strains requires further study.

## 5 Conclusions

Predictive model constructed using characteristic variables screened using LASSO regression analysis can accurately predict the prognosis of patients with severe COVID-19. This model can assist clinicians in implementing early interventions. Future prospective large-sample studies are required to validate our findings.

## Data availability statement

The original contributions presented in the study are included in the article/**Supplementary Material**. Further inquiries can be directed to the corresponding authors.

## Ethics statement

The studies involving humans were approved by the Biomedical Ethics Committee of the Fourth People’s Hospital of Shanghai (review and approval number: SYLL2023082). The studies were conducted in accordance with the local legislation and institutional requirements. Written informed consent for participation in this study was provided by the participants’ legal guardians/next of kin.

## Author contributions

XS: Data curation, Investigation, Writing – original draft. JT: Data curation, Investigation, Writing – original draft. JL: Project administration, Supervision, Writing – review & editing. HZ: Methodology, Supervision, Writing – review & editing. CL: Conceptualization, Funding acquisition, Writing – review & editing.
