# Supplementary material for: Development and validation of a prediction model for mortality in critically ill COVID-19 patients
Source: Front Cell Infect Microbiol. 2024 Jun 24;14:1309529. doi: 10.3389/fcimb.2024.1309529 (PMC11228157; doi:10.3389/fcimb.2024.1309529)
Supplement: Supplementary file 1 [file Table_1.docx]

Supplementary Material

Development and validation of a prediction model for mortality in critically ill COVID-19 patients

Xiaoxiao Sun†,1, Jinxuan Tang†,1, Jun Lu*,1, Hui Zhang*,1, Cheng Li*,1

*** Correspondence:**

Cheng Li, Tel: +86-021-55603999, Email: [chengli_2017@tongji.edu.cn](mailto:marklisa@163.com;)

Hui Zhang, Tel:+86-021-55603999, Email: [15077169795@163.com](mailto:15077169795@163.com)

Jun Lu, Tel: +86-021-55603999, Email: [smmu200709@aliyun.com](mailto:15077169795@163.com)

# Supplementary Tables

**e-Table 1. The characteristics of all patients admitted to ICU.**

| **Variables** | **Alive**  **(n = 84)** | **Dead**  **(n = 53)** | **P value** | **Variables** | **Alive**  **(n = 84)** | **Dead**  **(n = 53)** | **P value** |
| --- | --- | --- | --- | --- | --- | --- | --- |
| **Age** | 85.50  [73.00, 90.00] | 87.00  [76.00,91.00] | 0.229 | **PDW** |  |  | 0.379 |
| **Gender** |  |  | 0.691 | **15-17** | 76 (93.9) | 46 (86.8) |  |
| **Male** | 37 (44.0) | 26 (49.1) |  | **<15 / >17** | 6 (7.1) | 7 (13.2) |  |
| **Female** | 47 (56.0) | 27 (50.9) |  | **CRP** |  |  | 0.336 |
| **Hypertension** |  |  | 0.003 | **<=6** | 6 (7.1) | 1 (1.9) |  |
| **Yes** | 65 (77.4) | 27 (50.9) |  | **>6** | 78 (92.9) | 52 (98.1) |  |
| **No** | 19 (22.6) | 26 (49.1) |  | **Procalcitonin** |  |  | 0.003 |
| **Diabetes** |  |  | 0.014 | **< 0.5** | 56 (66.7) | 21 (39.6) |  |
| **Yes** | 24 (28.6) | 5 (9.4) |  | **>= 0.5** | 28 (33.3) | 32 (60.4) |  |
| **No** | 60 (71.4) | 48 (90.6) |  | **IL6** |  |  | 1.000 |
| **Apoplexy** |  |  | 0.031 | **<=6.6** | 2 (2.4) | 1 (1.9) |  |
| **Yes** | 44 (52.4) | 17 (32.1) |  | **>6.6** | 82 (97.6) | 52 (98.1) |  |
| **No** | 40 (47.6) | 36 (67.9) |  | **ALT** |  |  | 0.379 |
| **Coronary Disease** |  |  | 0.369 | **9-50** | 67 (79.8) | 38 (71.7) |  |
| **Yes** | 33 (39.3) | 16 (30.2) |  | **<9 / >50** | 17 (20.2) | 15 (28.3) |  |
| **No** | 51 (60.7) | 37 (69.8) |  | **AST** |  |  | <0.001 |
| **Mental diseases** |  |  | 0.804 | **15-40** | 62 (73.8) | 17 (30.2) |  |
| **Yes** | 7 (8.3) | 3 (5.7) |  | **<15 / >40** | 22 (26.2) | 37 (69.8) |  |
| **No** | 77 (91.7) | 50 (94.3) |  | **rGTT** |  |  | 0.642 |
| **Vaccination** |  |  | 0.527 | **10-60** | 69 (82.1) | 41 (77.4) |  |
| **0** | 78 (92.9) | 50 (94.3) |  | **<10 / >60** | 15 (17.9) | 12 (22.6) |  |
| **1** | 1 (1.2) | 0 (0.0) |  | **BUN** |  |  | 0.132 |
| **2** | 3 (3.6) | 3 (5.7) |  | **3.1-8.0** | 27 (32.1) | 10 (18.9) |  |
| **3** | 2 (2.4) | 0 (0.0) |  | **<3.1 / >8.0** | 57 (67.9) | 43 (81.1) |  |
| **WBC** |  |  | 0.470 | **Creatinine** |  |  | 0.007 |
| **3.5-9.5** | 35 (41.7) | 18 (44.0) |  | **57-97** | 30 (35.7) | 7 (13.2) |  |
| **<3.5/>9.5** | 49 (58.3) | 35 (66.0) |  | **<57 / >97** | 54 (64.3) | 46 (86.8) |  |
| **RBC** |  |  | 0.337 | **GFR** |  |  | 0.055 |
| **4.3-5.8** | 16 (19.0) | 6 (11.3) |  | **90-120** | 18 (21.4) | 5 (7.5) |  |
| **<4.3/>5.8** | 68 (81.0) | 47 (88.7) |  | **<90 / >120** | 66 (78.6) | 49 (92.5) |  |
| **Hb** |  |  | 0.464 | **Kalium** |  |  | <0.001 |
| **130-175** | 10 (13.1) | 4 (7.5) |  | **3.5-5.5** | 77 (91.7) | 35 (66.0) |  |
| **<130/>175** | 73 (86.9) | 49 (92.5) |  | **<3.5 / >5.5** | 7 (8.3) | 18 (34.0) |  |
| **Hct** |  |  | 0.269 | **Sodium** |  |  | 0.062 |
| **40-50** | 13 (15.5) | 4 (7.5) |  | **135-145** | 56 (66.7) | 26 (49.1) |  |
| **<40/>50** | 71 (84.5) | 49 (92.5) |  | **<135 / >145** | 28 (33.3) | 27 (50.9) |  |
| **MCV** |  |  | 0.275 | **Chlorine** |  |  | 0.306 |
| **82-100** | 75 (89.3) | 43 (81.1) |  | **96-108** | 50 (59.5) | 26 (49.1) |  |
| **<82/>100** | 9 (10.7) | 10 (18.9) |  | **<96/ >108** | 34 (40.5) | 27 (50.9) |  |
| **MCH** |  |  | 1.000 | **TnI** |  |  | 0.001 |
| **27-34** | 73 (86.9) | 46 (86.8) |  | **<0.028** | 36 (42.9) | 8 (15.1) |  |
| **<27/>34** | 11 (13.1) | 7 (13.2) |  | **>0.028** | 48 (57.1) | 45 (84.9) |  |
| **MCHC** |  |  | 0.305 | **Myoglobin** |  |  | 0.002 |
| **316-354** | 65 (77.4) | 36 (67.9) |  | **20.56-72.48** | 24 (28.6) | 3 (5.7) |  |
| **<316/>354** | 19 (22.6) | 17 (32.1) |  | **<20.56/ >72.48** | 60 (71.4) | 50 (94.3) |  |
| **PLT** |  |  | 0.807 | **CK_MB** |  |  | 0.005 |
| **125-350** | 60 (71.4) | 36 (67.9) |  | **<= 4.88** | 61 (72.6) | 25 (47.2) |  |
| **<125/>350** | 24 (28.6) | 17 (32.1) |  | **> 4.88** | 23 (27.4) | 28 (52.8) |  |
| **L** |  |  | 0.243 | **PT** |  |  | 0.044 |
| **1.1-3.2** | 21 (25.0) | 8 (15.1) |  | **9.4-12.5** | 54 (64.3) | 25 (45.3) |  |
| **<1.1/>3.2** | 63 (75.0) | 45 (84.9) |  | **<9.4/ >12.5** | 30 (35.7) | 29 (54.7) |  |
| **Monocytes** |  |  | 1.000 | **TT** |  |  | 1.000 |
| **0.1-0.6** | 59 (70.2) | 35 (69.8) |  | **13-21** | 72 (85.7) | 47 (86.8) |  |
| **<0.1/>0.6** | 25 (29.8) | 16 (30.2) |  | **<13/ >21** | 12 (14.3) | 7 (13.2) |  |
| **N** |  |  | 0.460 | **APTT** |  |  | 1.000 |
| **1.8-6.3** | 20 (23.8) | 9 (17.0) |  | **23.5-40.7** | 71 (84.5) | 44 (83.0) |  |
| **<1.8/>6.3** | 64 (76.2) | 44 (83.0) |  | **<23.5/ >40.7** | 13 (15.5) | 9 (17.0) |  |
| **RDW CV** |  |  | 0.906 | **Fibrinogen** |  |  | 1.000 |
| **11-16** | 71 (84.5) | 46 (86.8) |  | **2-5** | 63 (75.0) | 40 (75.5) |  |
| **<11/>16** | 13 (15.5) | 7 (13.2) |  | **<2/ >5** | 21 (25.0) | 13 (24.5) |  |
| **RDW SD** |  |  | 0.169 | **D2 dimer** |  |  | 0.689 |
| **37-50** | 60 (61.4) | 31 (58.5) |  | **< 0.5** | 2 (2.4) | 0 (0.0) |  |
| **<37/>50** | 24 (28.6) | 22 (41.5) |  | **> 0.5** | 82 (97.6) | 53 (100.0) |  |
| **MPV** |  |  | 0.666 |  |  |  |  |
| **8-12.5** | 71 (84.5) | 47 (88.7) |  |  |  |  |  |
| **<8/>12.5** | 13 (15.5) | 6 (11.3) |  |  |  |  |  |

(* *P* < 0.05)

**e-Table 2. The characteristics of patients after PSM.**

| **Variables** | **Alive**  **(n = 43)** | **Dead**  **(n = 43)** | **P value** | **Variables** | **Alive**  **(n = 43)** | **Dead**  **(n = 43)** | **P value** |
| --- | --- | --- | --- | --- | --- | --- | --- |
| **Age** | 87.00  [73.00, 90.00] | 87.00  [76.50, 90.50] | 0.346 | **PDW** |  |  | 0.737 |
| **Gender** |  |  | 1 | **15-17** | 39 (90.7) | 37 (86.0) |  |
| **Male** | 21 (48.8) | 21 (48.8) |  | **<15 / >17** | 4 (9.3) | 6 (14.0) |  |
| **Female** | 22 (51.2) | 22 (51.2) |  | **CRP** |  |  | 0.609 |
| **Hypertension** |  |  | 1 | **<=6** | 3 (7.0) | 1 (2.3) |  |
| **Yes** | 27 (62.8) | 27 (62.8) |  | **>6** | 40 (93.0) | 42 (97.7) |  |
| **No** | 16(37.2) | 16 (37.2) |  | **Procalcitonin** |  |  | 0.031 |
| **Diabetes** |  |  | 1 | **< 0.5** | 27 (62.8) | 17 (37.2) |  |
| **Yes** | 5 (11.6) | 5 (11.6) |  | **>= 0.5** | 16 (37.2) | 27 (62.8) |  |
| **No** | 38(88.4) | 38(88.4) |  | **IL6** |  |  | 1.000 |
| **Apoplexy** |  |  | 1 | **<=6.6** | 2 (4.7) | 1 (2.3) |  |
| **Yes** | 17 (39.5) | 17 (39.5) |  | **>6.6** | 41 (95.3) | 42 (97.7) |  |
| **No** | 27 (60.5) | 27(60.5) |  | **ALT** |  |  | 0.791 |
| **Coronary Disease** |  |  | 1 | **9-50** | 35 (81.4) | 33 (76.7) |  |
| **Yes** | 17 (39.5) | 17 (39.5) |  | **<9 / >50** | 8 (18.6) | 10 (23.3) |  |
| **No** | 27 (60.5) | 27(60.5) |  | **AST** |  |  | <0.001 |
| **Mental diseases** |  |  | 0.430 | **15-40** | 32 (74.4) | 13 (30.2) |  |
| **Yes** | 5 (11.6) | 2 ( 4.7) |  | **<15 / >40** | 11 (25.6) | 30 (69.8) |  |
| **No** | 38 (88.4) | 41 (95.3) |  | **rGTT** |  |  | 1.000 |
| **Vaccination** |  |  | 0.336 | **10-60** | 33 (76.7) | 34 (79.1) |  |
| **0** | 39 (90.7) | 41 (95.3) |  | **<10 / >60** | 10 (23.3) | 9 (20.9) |  |
| **1** | 1 (2.3) | 0 (0.0) |  | **BUN** |  |  | 0.132 |
| **2** | 1 (2.3) | 2 (4.7) |  | **3.1-8.0** | 14 (32.6) | 4 (16.3) |  |
| **3** | 2 (4.7) | 0 (0.0) |  | **<3.1 / >8.0** | 29 (67.4) | 36 (83.7) |  |
| **WBC** |  |  | 1.000 | **Creatinine** |  |  | 0.007 |
| **3.5-9.5** | 16 (37.2) | 15 (39.5) |  | **57-97** | 17 (39.5) | 5 (11.6) |  |
| **<3.5/>9.5** | 27 (62.8) | 26 (60.5) |  | **<57 / >97** | 26 (60.5) | 38 (88.4) |  |
| **RBC** |  |  | 0.256 | **GFR** |  |  | 0.052 |
| **4.3-5.8** | 10 (23.3) | 5 (11.6) |  | **90-120** | 12 (27.9) | 4 (9.3) |  |
| **<4.3/>5.8** | 33 (76.7) | 38 (88.4) |  | **<90 / >120** | 31 (72.1) | 39 (90.7) |  |
| **Hb** |  |  | 0.313 | **Kalium** |  |  | 0.022 |
| **130-175** | 7 (16.3) | 3 (7.0) |  | **3.5-5.5** | 77 (88.4) | 35 (65.1) |  |
| **<130/>175** | 36 (83.7) | 40 (93.0) |  | **<3.5 / >5.5** | 5 (11.6) | 15 (34.9) |  |
| **Hct** |  |  | 0.120 | **Sodium** |  |  | 0.280 |
| **40-50** | 10 (20.9) | 3 (7.0) |  | **135-145** | 26 (60.5) | 20 (46.5) |  |
| **<40/>50** | 34 (79.1) | 40 (93.0) |  | **<135 / >145** | 17 (39.5) | 23 (53.5) |  |
| **MCV** |  |  | 0.265 | **Chlorine** |  |  | 0.281 |
| **82-100** | 41 (95.3) | 37 (86.0) |  | **96-108** | 24 (55.8) | 18 (41.9) |  |
| **<82/>100** | 2 (4.7) | 6 (14.0) |  | **<96/ >108** | 19 (44.2) | 25 (58.1) |  |
| **MCH** |  |  | 0.710 | **TnI** |  |  | 0.008 |
| **27-34** | 38 (88.4) | 40 (93.0) |  | **<0.028** | 19 (41.9) | 6 (14.0) |  |
| **<27/>34** | 5 (11.6) | 3 (7.0) |  | **>0.028** | 25 (58.1) | 37 (86.0) |  |
| **MCHC** |  |  | 0.330 | **Myoglobin** |  |  | 0.004 |
| **316-354** | 34 (79.1) | 29 (67.4) |  | **20.56-72.48** | 15 (34.9) | 3 (7.0) |  |
| **<316/>354** | 9 (20.9) | 14 (32.6) |  | **<20.56/ >72.48** | 28 (65.1) | 40 (93.0) |  |
| **PLT** |  |  | 0.818 | **CK_MB** |  |  | 0.079 |
| **125-350** | 30 (69.8) | 28 (65.1) |  | **<= 4.88** | 30 (69.8) | 21 (48.8) |  |
| **<125/>350** | 13 (30.2) | 15 (34.9) |  | **> 4.88** | 13 (30.2) | 22 (51.2) |  |
| **L** |  |  | 0.381 | **PT** |  |  | 0.388 |
| **1.1-3.2** | 9 (20.9) | 5 (11.6) |  | **9.4-12.5** | 24 (55.8) | 19 (44.2) |  |
| **<1.1/>3.2** | 34 (79.1) | 38 (88.4) |  | **<9.4/ >12.5** | 19 (44.2) | 24 (55.8) |  |
| **Monocytes** |  |  | 1.000 | **TT** |  |  | 0.756 |
| **0.1-0.6** | 33 (76.7) | 32 (74.4) |  | **13-21** | 36 (83.7) | 39 (88.4) |  |
| **<0.1/>0.6** | 10 (23.3) | 11 (25.6) |  | **<13/ >21** | 7 (16.3) | 5 (11.6) |  |
| **N** |  |  | 0.782 | **APTT** |  |  | 1.000 |
| **1.8-6.3** | 9 (20.9) | 7 (17.0) |  | **23.5-40.7** | 37 (86.0) | 38 (88.4) |  |
| **<1.8/>6.3** | 34 (79.1) | 36 (83.7) |  | **<23.5/ >40.7** | 6 (14.0) | 5 (11.6) |  |
| **RDW CV** |  |  | 0.710 | **Fibrinogen** |  |  | 0.805 |
| **11-16** | 40 (93.0) | 38 (88.4) |  | **2-5** | 31 (72.1) | 33 (76.7) |  |
| **<11/>16** | 3 (7.0) | 5 (11.6) |  | **<2/ >5** | 12 (27.9) | 10 (23.3) |  |
| **RDW SD** |  |  | 0.038 | **D-dimer** |  |  | 1.000 |
| **37-50** | 34 (79.1) | 24 (54.8) |  | **< 0.5** | 0 (0.0) | 0 (0.0) |  |
| **<37/>50** | 9 (20.9) | 19 (44.2) |  | **> 0.5** | 43 (100.0) | 43 (100.0) |  |
| **MPV** |  |  | 0.770 |  |  |  |  |
| **8-12.5** | 35 (81.4) | 37 (86.0) |  |  |  |  |  |
| **<8/>12.5** | 8 (18.6) | 6 (14.0) |  |  |  |  |  |

(* *P* < 0.05)
